# Supplementary material for: The effect of fertility on female labour force participation in Tanzania
Source: PLoS One. 2024 Jan 2;19(1):e0292122. doi: 10.1371/journal.pone.0292122 (PMC10760919; doi:10.1371/journal.pone.0292122)
Supplement: S1 Appendix — (DOCX) [file pone.0292122.s001.docx]

**Appendix 1:** Econometric Results

| **Variable name** |  | **Coefficient** | | | | **Marginal effect** | | | |
| --- | --- | --- | --- | --- | --- | --- | --- | --- | --- |
|  |  | Probit | IV probit | 2SRI | CFA | Probit | IV probit | 2SRI | CFA |
| Fertility |  | -0.039*** | -0.130*** | -0.126*** | -0.126*** | -0.011*** | -0.130*** | -0.034*** | -0.035*** |
|  |  | (0.01) | (0.041) | (0.039) | (0.039) | (0.003) | (0.041) | (0.011) | (0.011) |
| Education | Primary | 0.144*** | 0.092* | 0.094* | 0.092* | 0.041*** | 0.092* | 0.026* | 0.025* |
|  |  | (0.044) | (0.05) | (0.049) | (0.049) | (0.013) | (0.05) | (0.014) | (0.014) |
|  | Secondary | 0.105* | 0.011 | 0.012 | 0.008 | 0.028* | 0.011 | 0.003 | 0.003 |
|  |  | (0.06) | (0.072) | (0.072) | (0.072) | (0.016) | (0.072) | (0.019) | (0.019) |
|  | Higher | 0.668*** | 0.541** | 0.561** | 0.555** | 0.134*** | 0.541** | 0.115*** | 0.113*** |
|  |  | (0.191) | (0.232) | (0.228) | (0.228) | (0.025) | (0.232) | (0.033) | (0.034) |
| Husband employment | Employee | 0.07 | -0.005 | 0.003 | 0.003 | 0.019 | -0.005 | 0.001 | -0.002 |
|  |  | (0.141) | (0.15) | (0.15) | (0.15) | (0.039) | (0.15) | (0.041) | (0.041) |
|  | Self-empl | 0.481*** | 0.410*** | 0.413*** | 0.412*** | 0.134*** | 0.410*** | 0.112*** | 0.113*** |
|  |  | (0.141) | (0.15) | (0.15) | (0.15) | (0.039) | (0.15) | (0.041) | (0.041) |
| Contraceptive use |  | 0.252*** | 0.244*** | 0.251*** | 0.249*** | 0.068*** | 0.244*** | 0.066*** | 0.065*** |
|  |  | (0.036) | (0.036) | (0.037) | (0.037) | (0.009) | (0.036) | (0.009) | (0.009) |
| Wealth | Richest | -0.066 | -0.127 | -0.137 | -0.14 | -0.019 | -0.127 | -0.038 | -0.036 |
|  |  | (0.073) | (0.085) | (0.087) | (0.087) | (0.021) | (0.085) | (0.025) | (0.024) |
|  | Richer | -0.141** | -0.136** | -0.147** | -0.148** | -0.041** | -0.136** | -0.041** | -0.038** |
|  |  | (0.058) | (0.062) | (0.063) | (0.063) | (0.017) | (0.062) | (0.018) | (0.018) |
|  | Mid | 0.04 | 0.044 | 0.039 | 0.038 | 0.011 | 0.044 | 0.01 | 0.012 |
|  |  | (0.056) | (0.058) | (0.059) | (0.058) | (0.015) | (0.058) | (0.016) | (0.015) |
|  | Poorer | 0.203*** | 0.214*** | 0.207*** | 0.207*** | 0.053*** | 0.214*** | 0.053*** | 0.055*** |
|  |  | (0.057) | (0.059) | (0.059) | (0.059) | (0.014) | (0.0059) | (0.014) | (0.014) |
| Married |  | -0.270*** | -0.273*** | -0.251*** | -0.248*** | -0.071*** | -0.273*** | -0.064*** | -0.070*** |
|  |  | (0.039) | (0.042) | (0.044) | (0.044) | (0.01) | (0.042) | (0.01) | (0.01) |
| Rural |  | 0.163*** | 0.192*** | 0.189*** | 0.191*** | 0.047*** | 0.192*** | 0.053*** | 0.055*** |
|  |  | (0.046) | (0.049) | (0.049) | (0.049) | (0.014) | (0.049) | (0.014) | (0.014) |
| Age |  | 0.108*** | 0.146*** | 0.142*** | 0.142*** | 0.030*** | 0.146*** | 0.038*** | 0.040*** |
|  |  | (0.015) | (0.021) | (0.02) | (0.02) | (0.004) | (0.021) | (0.006) | (0.006) |
| Age square |  | -0.001*** | -0.001*** | -0.001*** | -0.001*** | 0.000*** | -0.001*** | 0.000*** | 0.000*** |
|  |  | (0.000) | (0.000) | (0.000) | (0.000) | (0.000) | (0.000) | (0.000) | (0.000) |
| Household size |  | 0.015*** | 0.025*** | 0.024*** | 0.025*** | 0.004*** | 0.025*** | 0.007*** | 0.007*** |
|  |  | (0.005) | (0.007) | (0.007) | (0.007) | (0.001) | (0.007) | (-0.002) | (0.002) |
| uhat_fert |  |  |  | 0.096** | 0.112** |  |  | 0.026** | 0.031** |
|  |  |  |  | (-0.04) | (-0.044) |  |  | (0.011) | (0.012) |
| fert_inter |  |  |  |  | -0.003 |  |  |  | -0.001 |
|  |  |  |  |  | (0.003) |  |  |  | (0.001) |
| Number of observations |  | 8189 | 7618 | 7618 | 7618 |  |  |  |  |
| Wald chi2(16) |  | 593.51 | 522.01 | 501.63 | 501.18 |  |  |  |  |
| Prob > chi2 |  | 0.000 | 0.000 | 0.000 | 0.000 |  |  |  |  |
| Pseudo R2 |  | 0.078 |  | 0.072 | 0.072 |  |  |  |  |
| Log pseudo likelihood |  | -3943.912 | -18078.7 | -3602.66 | -3602.29 |  |  |  |  |
| Wald test of exogeneity (corr = 0): χ^2^(1) = 5.39 Prob > χ^2^ = 0.0203 | | | |  |  |  |  |  |  |

***, **, * represents significance level at 1%, 5% and 10% respectively, Standard errors in parentheses
