# Supplementary material for: The effect of fertility on female labour force participation in Tanzania
Source: PLoS One. 2024 Jan 2;19(1):e0292122. doi: 10.1371/journal.pone.0292122 (PMC10760919; doi:10.1371/journal.pone.0292122)
Supplement: Attachment — Submitted filename: Responses to Reviewers comments.docx [file pone.0292122.s002.docx]

Reviewers' comments:

Reviewer's Responses to Questions

**Comments to the Author**

1. Is the manuscript technically sound, and do the data support the conclusions?

The manuscript must describe a technically sound piece of scientific research with data that supports the conclusions. Experiments must have been conducted rigorously, with appropriate controls, replication, and sample sizes. The conclusions must be drawn appropriately based on the data presented.

Reviewer #1: Yes

Reviewer #2: Partly

2. Has the statistical analysis been performed appropriately and rigorously?

Reviewer #1: No

Reviewer #2: No

3. Have the authors made all data underlying the findings in their manuscript fully available?

The [PLOS Data policy](http://www.plosone.org/static/policies.action#sharing) requires authors to make all data underlying the findings described in their manuscript fully available without restriction, with rare exception (please refer to the Data Availability Statement in the manuscript PDF file). The data should be provided as part of the manuscript or its supporting information, or deposited to a public repository. For example, in addition to summary statistics, the data points behind means, medians and variance measures should be available. If there are restrictions on publicly sharing data—e.g. participant privacy or use of data from a third party—those must be specified.

Reviewer #1: No

Reviewer #2: Yes

4. Is the manuscript presented in an intelligible fashion and written in standard English?

PLOS ONE does not copyedit accepted manuscripts, so the language in submitted articles must be clear, correct, and unambiguous. Any typographical or grammatical errors should be corrected at revision, so please note any specific errors here.

Reviewer #1: Yes

Reviewer #2: Yes

5. Review Comments to the Author

Please use the space provided to explain your answers to the questions above. You may also include additional comments for the author, including concerns about dual publication, research ethics, or publication ethics. (Please upload your review as an attachment if it exceeds 20,000 characters)

Reviewer #1: This study examines a very interesting and valuable topic and applies an IV method to tackle the endogeneity issue of fertility decisions.

1. However, I found the discussion of IV validity in general missing. Among the instruments, the subjective instrument -- Mother’s opinion on the ideal family size -- can especially be argued to be endogeneous as women's personality or preference directly impacts both their fertility and labor supply decisions, which violates the validity assumption. Performing the overidentication test would help provide inferences for the validity of instruments. Reporting weak identification test results is also standard while using multiple IVs, although first-stage F statistics provide references for instrument relevance. Overall, IVs deserve more careful analyses.

**RESPONSES**:

The discussion of IV validity has been added and improved as per suggestion (see section 4.2 and table 3). Also, the justification for the use of Mother’s opinion on the ideal family size has been justified (the last paragraph of section 3.2, line xxx). In addition, the test of relevance and validity of the instruments was done (table 3) and all the instruments were valid and relevant, hence Mother’s opinion on the ideal family size had no influence on labour supply decisions except through fertility.

2. Regarding the analytical framework, I found the notation inconsistent and confusing sometimes. Among others, C sometimes denotes time allocated to work, sometimes means consumption.

**RESPONSES:**

The letters have been changed to avoid confusion. Letter C now denotes Consumption whereas time is denoted by letter T.

3.Moreover, the analytical framework does not add much to the discussion nor guide the empirical analysis. Latent variable is discussed but not applied in empirical model -- it may make a difference if female labor supply at the intensive margin is studied as an outcome variable.
**RESPONSES:**

Analytical framework has been improved and more description on the methods used has been added (See section 3.1). The latent variable explained in the study represents an unobservable that implies the likelihood of females being in the labour market. Therefore, the dependent variable in this case labour force participation has been studied as an outcome variable as described from equation 12-18.

4. As briefly discussed by the authors in the paper, some chosen IVs only work for women with at least two children. Would including and excluding these IVs affect the sample size and your results differently? What is the proportion of women with at least two children? Some descriptive statistics would help.

**RESPONSES:**

Excluding the instrumental variables affects the sample sizes and results differently. By including the instrumental variables (multiple first birth and the sex composition of first two children) the number of observations reduces from 13,266 to 9,720. However, excluding them will lead to biased estimates as the endogeneity of fertility in the labour force participation equation will not be accounted. Additionally, the descriptive statistics of women with at least two children has been added.

Reviewer #2: This paper studies the link between female labour force participation (LFP) and fertility in Tanzania in 2015-2016 period. The empirical strategy exploits cross-sectional variation in fertility and finds that additional children reduce female labour force participation. This is consistent with prior literature documenting similar relationship.

My comments below mostly focus on framing the paper's contribution, and are presented in the order they came up as I read:

1. I wonder what is particular about the Tanzanian context that would motivate investigating the relationship only there, compared to other African contexts. Even if female LFP is the highest there as depicted in Figure 1, this doesn't mean the relationship between fertility and LFP is quite particular to Tanzania. I think it would be more interesting and much more enriching to see a replication of same exercise in other African countries.
**RESPONSES:**

The justification for the inclusion of Tanzania as the case study has been added with relevant literature. As stated in the last paragraph of section 2, while there are a number of studies regarding fertility and labour force participation in many African countries, in Tanzania such studies are lacking. This is despite the fact that Tanzania is among the countries with very high fertility and labour force participation rates in the world (see figure 1).

2. I think the "related literature" section could be more clear about what the exact contribution of the paper is. So, it would be helpful for framing the paper's contribution more clearly (is the contribution of this paper causal identification of the relationship between fertility and LFP, and/or is the contribution the setting - in Tanzania?). Have there been other papers that have examined the same relationship in Africa? What were their results?

**RESPONSES:**

The contribution of the paper has been improved in the relevant literature section. In addition, more discussion on the contribution of the study in relation to other studies in the African region has been added. As stated in the last paragraph of section 2, the study is to the best of the researchers’ knowledge; the first to examine the causal effect of fertility and LFP in Tanzania.

3. It would be helpful to provide some graphical evidence before looking at regressions. For example, a graph of say, mean children ever born and average female LFP in given province/district.

**RESPONSES:**

The summary of the information suggested have been presented in table 2

4. Data: is there only 1 DHS survey for Tanzania? This makes a big limitation for the study, by not being able to track the relationship over time. Is it possible to supplement these results with other survey data from Afrobarometer and other World Bank household surveys?

**RESPONSES:**

The DHS survey used herewith is the latest public available dataset in Tanzania. Afrobarometer and World bank data had some inconsistency in terms of specific variables of interest. However, we are planning to write a follow up paper by integrating the upcoming Tanzania DHS that might be released by the government in future and we might use different approaches (in terms of the methodologies).

5. As a result, the empirical specification would follow a cross section design, with inability to control for province or district fixed effects, since there is no time variation. The use of IV is quite important to control for reverse feedback and other unobservables, but then the author mentioned it used "two stage residual inclusion function approach" without justifying its importance and how it improves upon profit IV. All methodologies used to estimate the relationship should be carefully elaborated in the empirical specification section. There is also no discussion on the exclusion restriction of IV.

**RESPONSES:**

The empirical specification section has been improved. Specifically, the use of the "two stage residual inclusion and the control function approach" has also been justified

6. Results in Table 4 are quite puzzling especially with regards to magnitudes. In column 2, the estimated coefficient of the IV is quite very large compared to OLS, I wonder why? No explanation was given. I also don't understand the point of using two stage residual inclusion in column 3 and non-linear interactions of fertility with unobservables as shown in column 4. How this improves upon profit IV, which is supposed to correct for non-observables by default.

**RESPONSES:**

7. Also, based on table 2 of descriptive statistics, I see we have an unbalanced dataset. I wonder if the addition of controls led to a drop of sample size and affected the estimated coefficients. What would happen if we estimated the equation without controls?

**RESPONSES:**

We tested for the same and found insignificant results without control

8. How about non-linear relationship? I would like to see interactions of the fertility variable with individual characteristics to explore their conditional effects.

**RESPONSES:**

We tested for non-linearity relationship and the results were not significant.

Minor comments:

1- Results table must include the number of observations for each model and adjusted R-squared to evaluate the predicative power of the model.

**RESPONSES**

The total number of observations has been indicated in Table 2 i.e. 13,266.

2- A short paragraph should be included in the introduction that defines the sections of the paper.

**RESPONSES**

The comments have been addressed based on reviewer’s suggestions. A paragraph on the introduction of the paper’s sections has been added. ‘The rest of the paper is structured with the following sections; relevance to the literature, methodology, results and conclusions’.
